# Diagnostic Performance of Parasitological, Immunological, Molecular, and Ultrasonographic Tests in Diagnosing Intestinal Schistosomiasis in Fieldworkers From Endemic Municipalities in the Philippines

**DOI:** 10.3389/fimmu.2022.899311

**Published:** 2022-06-14

**Authors:** Ian Kim B. Tabios, Marcello Otake Sato, Ourlad Alzeus G. Tantengco, Raffy Jay C. Fornillos, Masashi Kirinoki, Megumi Sato, Raniv D. Rojo, Ian Kendrich C. Fontanilla, Yuichi Chigusa, Paul Mark B. Medina, Mihoko Kikuchi, Lydia R. Leonardo

**Affiliations:** ^1^ Institute of Biology, College of Science, University of the Philippines Diliman, Quezon City, Philippines; ^2^ College of Medicine, University of the Philippines Manila, Manila, Philippines; ^3^ Laboratory of Tropical Medicine and Parasitology, Dokkyo Medical University, Tochigi, Japan; ^4^ Graduate School of Health Sciences, Niigata University, Niigata City, Japan; ^5^ Center for International Cooperation, Dokkyo Medical University, Tochigi, Japan; ^6^ Department of Biochemistry and Molecular Biology, College of Medicine, University of the Philippines Manila, Manila, Philippines; ^7^ Institute of Tropical Medicine, Nagasaki University, Nagasaki, Japan; ^8^ Office of Research Coordination, University of the East, Manila, Philippines; ^9^ College of Arts and Sciences, University of the Philippines Manila, Manila, Philippines; ^10^ University of the East Ramon Magsaysay Graduate School, Quezon City, Philippines

**Keywords:** *Schistosoma japonicum*, immunodiagnosis, LAMP, PCR, ultrasound, positivity

## Abstract

Schistosomiasis remains to ha/ve a significant public health impact in the Philippines. The Kato-Katz (K-K) technique is the reference standard and most used technique for definitive diagnosis of intestinal schistosomiasis for control programs in endemic regions. However, this has a very low sensitivity when applied in areas of low endemicity and patients with light infection. Hence, this study determined the diagnostic performance of immunological, molecular, parasitological, and ultrasonographic tests in diagnosing intestinal schistosomiasis in endemic municipalities in the Philippines. We performed a community-based cross-sectional study to determine the positivity of schistosomiasis in Leyte, Philippines. The diagnostic performance of five different detection techniques: (1) three stool K-K with duplicate smears; (2) soluble egg antigen IgG ELISA; (3) urine point-of-care circulating cathodic antigen (POC-CCA) test; (4) detection of *Schistosoma japonicum* circulating DNA (SjcDNA) in serum and urine samples; (5) focused abdominal ultrasound (US), were also obtained in this study. Multiple stool examinations enhanced the sensitivity of K-K from 26.2% (95% CI [16.4, 38.8]) with single stool to 53.8% (95% CI [41.1, 66.1]) and 69.2% (95% CI [56.4, 80.0]) with two and three stools from consecutive days, respectively. Among the SjcDNA nucleic acid amplification test (NAAT)-based detection assays, loop-mediated isothermal amplification (LAMP) PCR using sera had the highest sensitivity at 92.3% (95% CI [82.2, 97.1]) with LAMP consistently identifying more positive cases in both serum and urine samples. This study showed that single stool K-K, which remains the only diagnostic test available in most endemic areas in the Philippines, had low sensitivity and failed to identify most patients with light infection. SjcDNA detection assay and POC-CCA urine test were more sensitive than stool microscopy in detecting schistosomiasis. On the other hand, US was less sensitive than the widely utilized K-K technique in diagnosing schistosomiasis. This study emphasizes the need to revisit the use of single stool K-K in the surveillance and case detection of schistosomiasis in endemic areas of the Philippines. The availability of advanced and more sensitive diagnostic tests will help better control, prevent, and eliminate schistosomiasis in the country.

## Introduction

Intestinal schistosomiasis caused by *Schistosoma japonicum* is a zoonotic disease with a significant public health impact in the Philippines. This neglected tropical disease is endemic in 28 of 81 provinces distributed in 12 of 18 regions (1). Approximately 12 million in 1,593 barangays are at risk of infection, with 2.5 million directly exposed to 3,012 snail-infested bodies of water, making *S. japonicum* a serious national public health problem (2). Most transmission foci are in Mindanao, particularly in the CARAGA region (3). There are 3,012 identified snail-infested bodies of water as of 2015, and 80% of these are in Mindanao, 18% in the Visayas, and only 2% in Luzon.

The reference standard and most used technique for definitive diagnosis of intestinal schistosomiasis for control programs in endemic regions is still the Kato-Katz (K-K) technique due to its simplicity and high specificity when performed by trained personnel (4, 5). However, microscopy-based techniques that detect parasite eggs in stools, like the K-K technique, have very low sensitivity when applied in regions of low endemicity and in patients with light infection (6, 7). In areas where yearly mass drug administration has been implemented, such as in endemic barangays in the Philippines, infection intensity is decreasing, with light infections (egg per gram [EPG] < 99) being more common (8). These patients with low egg count can be misdiagnosed. Hence no appropriate curative treatment is given (6, 7). Due to the low sensitivity of one stool thick smear K-K for light infections, three consecutive day stool K-K has been recommended but with limited acceptance due to the burden of collecting multiple fecal samples.

Accurate, cost-effective, and easy-to-use diagnostic tests are crucial in controlling and eliminating schistosomiasis (9). Several diagnostic techniques have been developed to improve the detection of schistosomiasis, especially in endemic areas (10). These diagnostic tests include soluble egg antigen (SEA) IgG ELISA, urine point-of-care circulating cathodic antigen (POC-CCA) test, and detection of *Schistosoma japonicum* circulating DNA (SjcDNA) in serum and urine samples using (NAAT)-based detection assays and loop-mediated isothermal amplification (LAMP) (5, 9–12). The utilization of most of these newer tests can give better results, but large-scale antigen production is still difficult (13, 14). PCR-based tests gained popularity due to their high degree of sensitivity and specificity. Previous studies have shown the utility of this test in detecting cell-free DNA in serum, urine, or saliva for *S. mansoni, S. haematobium*, and *S. japonicum* (9, 12). POC-CCA urine test has been validated in multiple African countries to diagnose *S. mansoni*, and the assay’s diagnostic performance greatly varies with sensitivities ranging from 37% to 98% (15–17).

Aside from molecular and immunologic tests, focused abdominal ultrasound (US) was also helpful in diagnosing patients with sub-clinical hepatosplenic disease due to chronic schistosomiasis. This complication of schistosomiasis remains prevalent in endemic areas of the Philippines despite decreasing reports of schistosomiasis-related morbidity and mortality (18). The low sensitivity of the diagnostic test for schistosomiasis being used in endemic regions of the Philippines hinders the progress of the control and elimination program (19). It results in the persistence of sub-clinical hepatosplenic manifestations and low-intensity infections. With the national target of interrupting transmission by 2025, there is a need for more current and accurate diagnostic tests for diagnosing schistosomiasis in the Philippines. This study determined the positivity rate of intestinal schistosomiasis caused by S. japonicum in endemic places in Leyte, Philippines using orthogonal tests, including parasitological, immunological, molecular, and ultrasonographic tests.

## Materials and Methods

### Ethical Considerations

This study was approved by the University of the Philippines Manila Research Ethics Board UPMREB Code 2017-369-01. Written informed consent was obtained from all participants at the beginning of the study.

### Study Location

**Figure 1:**
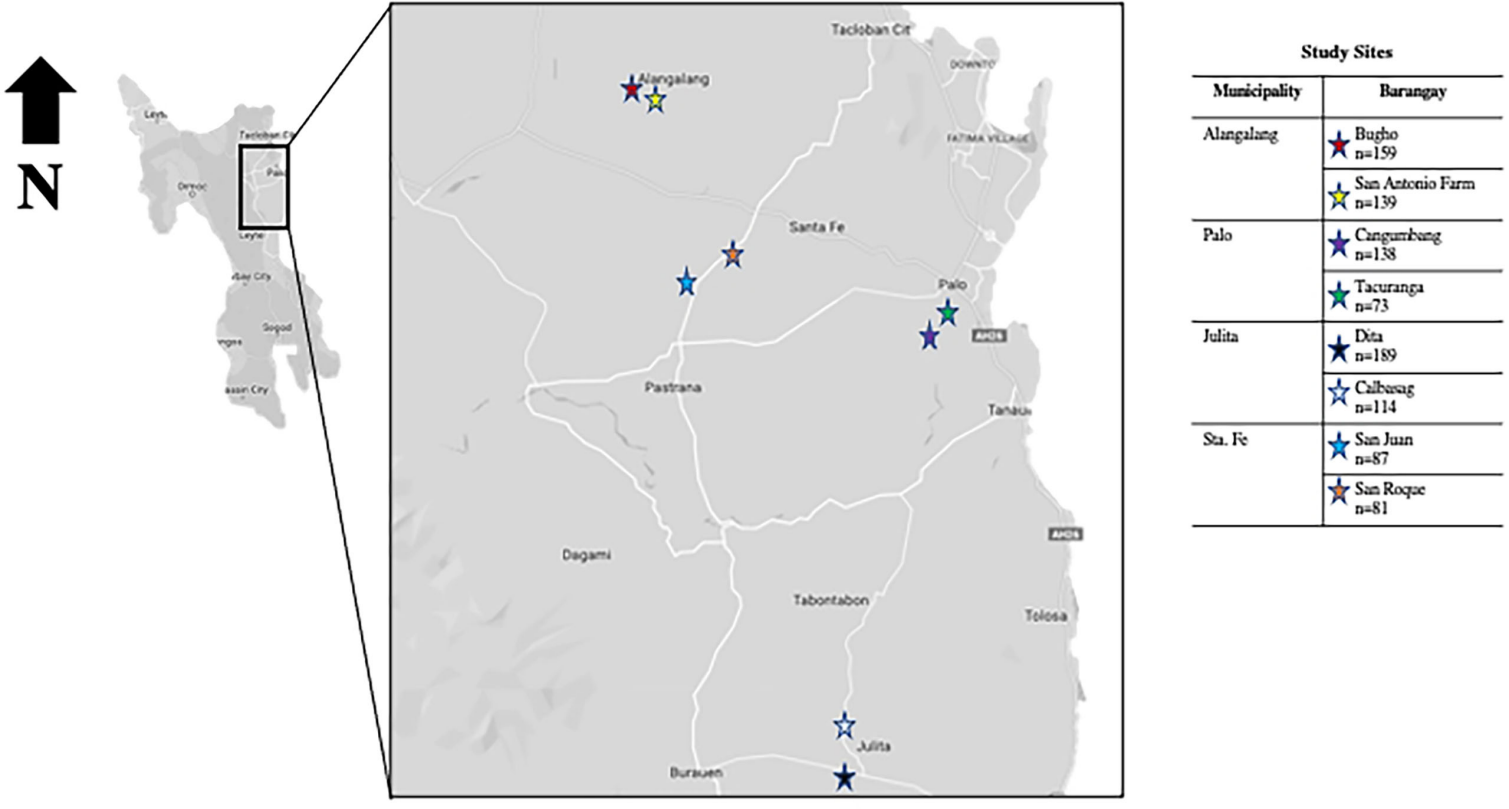
Map showing the study sites in the schistosomiasis endemic island of Leyte, Philippines.

The study sites comprised eight schistosomiasis endemic barangays (the smallest administrative division in the Philippines) in the municipalities of Julita, Alang-alang, Palo, and Sta Fe in the province of Leyte (**Figure 1**). Schistosomiasis classification of the selected barangays in these municipalities was based on the 2013-2015 single stool sentinel survey performed by the Department of Health Region VIII. The estimated prevalence of schistosomiasis using single stool duplicate Kato-Katz thick smear based on a focal survey in 2015 was 6.01% for Alangalang (8). These municipalities have had active schistosomiasis control programs with annual mass drug administration (MDA) with praziquantel for consenting individuals aged 5-65 years old since 2008 (20, 21).

### Study Design and Sampling Technique

Community-based cross-sectional study design and convenience sampling techniques were employed. Residents who were conveniently available during the fieldwork were invited to the study. All consenting individuals who submitted the required specimens were included in the study.

### Eligibility Criteria

The inclusion criteria for the initial cross-sectional survey were the following: (1) age 18 to 49 years old; (2) resides in the study area for more than five years based on the interview; (3) presence of significant exposure to potentially contaminated water based on the interview; and (4) agrees to participate in the study with signed informed consent forms. Individuals with these characteristics were excluded: (1) history of hepatitis virus types B and C based on the interview; (2) severely underweight (BMI < 16.00) or extremely obese (BMI > 40.0); (3) diagnosed with other hepatic disease based on the interview; (4) known case of malignancy based on the interview; (5) pregnant and/or currently lactating based on the interview; and (6) ≥ 60 grams per day of ethanol consumption for at least ten years based on interview.

### Participant Selection

**Figure 2:**
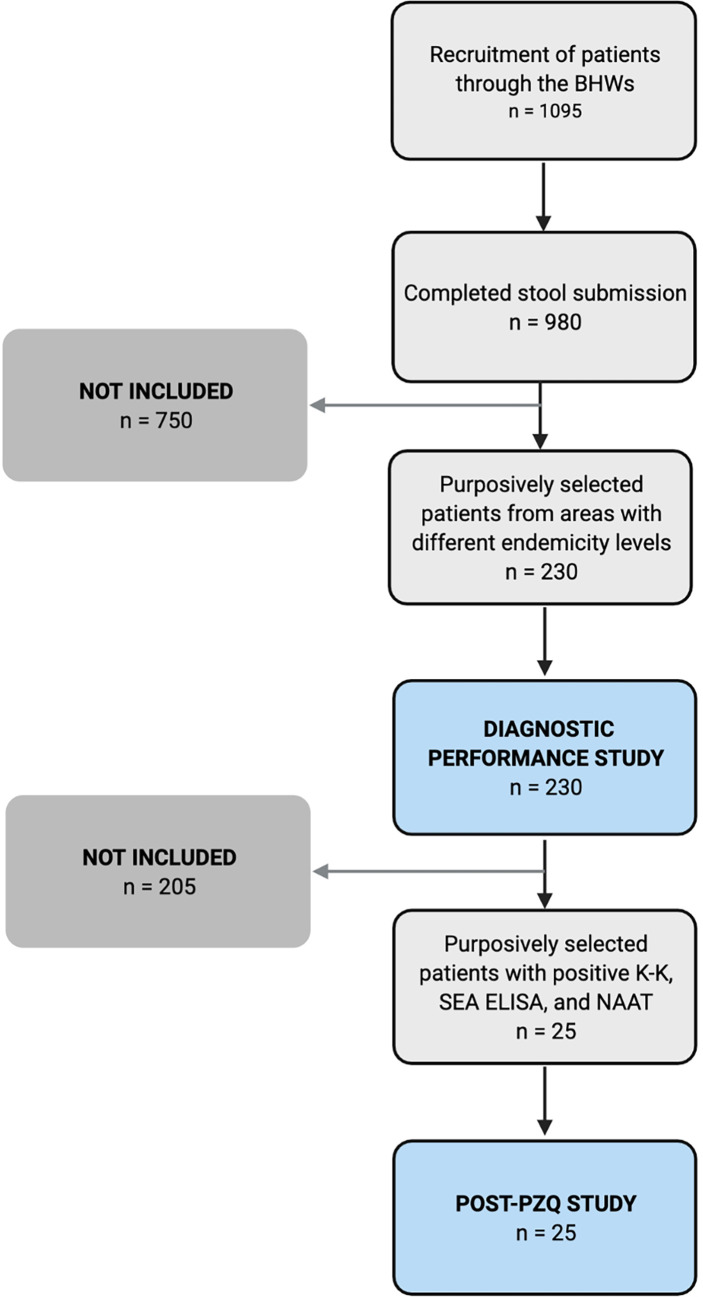
Recruitment of participants for the diagnostic performance (n = 230) and post-treatment studies (n = 25).

One thousand ninety-five (1095) residents showed interest to participate, of which 980 completed the three-stool submission. Individuals who tested positive in at least one of the three stool K-K were given treatment (PZQ 60 mg/kg in two divided doses at least 4 hours apart) under the supervision of the MHO or other attending physician. All 980 patients who completed the three-stool submission were included in the positivity survey. For the diagnostic performance study, 230 of the 980 patients were conveniently selected from three barangays with different endemicity levels. Of the 45 stool-positive cases in the 230 patients, 25 were conveniently selected for a post-PZQ follow-up study (**Figure 2**).

### Collection and Transport of Samples

Clean stool and urine containers were provided to participants. Three stool samples from three consecutive days were collected. Duplicate K-K thick smears per stool sample were immediately prepared following standard protocol and stored in sealed containers. A licensed medical technologist or trained phlebotomist performed venipuncture of the superficial veins of the upper limb. A strict aseptic technique was followed during blood extraction. At least 8-10 mL of blood were collected and transferred in 10 mL BD Vacutainer^®^ Blood Collection Tubes with a red stopper. Blood samples were left at room temperature for 1 hour and were then centrifuged at 4,000 rpm for 10 minutes at 4°C. This was followed by another centrifugation for 10 min at 10,000 rpm at 4°C. Hemolyzed samples were excluded, and blood extraction was repeated to obtain a new specimen. Equal amounts of serum were carefully transferred into three RNase-free 2 mL tubes and stored in 4°C. Urine (3 mL) was concentrated to 140 µL using an Amicon^®^ Ultra-15 Centrifugal Filter Device, 100K (Merck Millipore Ltd., Ireland). The urine concentrates were stored in 4°C inside sealed containers. Serum and urine samples were transported on ice. The universal precaution was done in handling the specimens. Materials like tubes and pipette tips used to evaluate the collected blood, serum, or urine were disinfected first with 0.5% hypochlorite solution, autoclaved, and disposed of as infectious waste material. Transfer of materials strictly followed regulations in the areas of origin and destination.

### Schistosomiasis Diagnostic Tests

In this study, the composite reference standard or the true positives (TP) were defined as being positive in at least one of the following tests: three stool K-K (3 K-K) or detecting *S. japonicum* in at least one smear from the three stools submitted, serum LAMP (sLAMP), urine LAMP (uLAMP), serum PCR (sPCR), and urine PCR (uPCR). A true negative (TN) was designated negative in all the specified tests. Blood extraction and clinical and ultrasonographic evaluation of the liver and spleen were performed during day 1 of stool submission.

#### Stool Examination

Three consecutive day stool K-K with duplicate thick smears were prepared. Trained microscopists performed the duplicate examination of three stool specimens by K-K technique. Schistosomiasis infection intensity was estimated based on WHO criteria, namely low, moderate, and high-intensity infections defined as egg per gram of stool (EPG) of 1-99, 100-399, and ≥ 400, respectively (22). EPG was obtained by multiplying by 24 the number of egg found in the fecal smear. Eggs of other helminths were noted. As part of quality control, 10% of all slides were re-examined in a separate laboratory at the University of the Philippines Manila.

#### SEA IgG ELISA

Standard procedure for SEA ELISA was performed with slight modifications (23, 24). Anti-Human IgG-peroxidase antibody produced in rabbit and 3,3′,5,5′-tetramethylbenzidine (KPL, Gaithersburg, MD) served as the secondary antibody and substrate, respectively. Polystyrene 96-well ELISA plates (Greiner Bio-One, Co., Ltd., Germany) was sensitized separately with SEA (1µg/well). Antigen was diluted with 0.05M carbonate bicarbonate buffer (pH 9.6). After blocking with 1% bovine serum albumin (BSA) in phosphate-buffered saline with 0.05% Tween 20 (T-PBS) (T-PBS-1%BSA), serum samples were distributed on the antigen-coated wells. Human sera (0.1 mL) and secondary antibody (0.1 mL) was diluted 200-fold and 10,000-fold, respectively. Optical density (OD) at 450 nm was measured using a microplate reader. Each ELISA reaction utilized positive (8-weeks post-infected rabbit serum) and negative (diluting buffer) controls. Triplicates were performed. Mean absorbance of sera of healthy controls plus 3 standard deviations was used as cut-off value. Samples with mean absorbance higher than the cut-off value were considered positive.

#### Urine POC-CCA

The WHO NTD Department provided POC-CCA kit. Urine was subjected to POC-CCA following the manufacturer’s instructions (Rapid Medical Diagnostics; Pretoria, South Africa). Briefly, a drop of urine was applied to the circular well in the test cassette, followed by a drop of the buffer. Two blinded observers read the test 20 minutes after adding the buffer. The presence of pink bands in both the test and control areas indicated a positive result. A faint band in the test area of the urine kit was noted and considered positive.

#### SjcDNA Detection in Serum and Urine Samples

Total DNA from urine was extracted using DNeasy Tissue Kit (QIAGEN Inc., Valencia, CA) according to the manufacturer’s protocol. DNA extracts were stored at -20°C until use. The primer pair CF (5’-GATCGTAAATTTGGA/TACTGC-3’) and CR (5’-CCAACCATAAACATATGATG-3’) specific for *S. japonicum* mitochondrial cytochrome oxidase subunit 1 gene was used. The final PCR mixture composed of 1.0 μL DNA template, 13.8 μL distilled water, 2.0 μL of buffer, 1.6 μL of dNTP, 0.6 μL of 1.5 mM MgCl_2_, 0.2 μL of 5 U/mL Taq DNA Polymerase (Takara, Otsu, Japan), and 0.5 μM each of forward and reverse primers. Standard PCR was done in an automated thermal cycler. PCR tubes were incubated for 40 cycles with the following programmed profile: initial denaturation for 10 min at 95°C and 40 cycles of amplification (denaturation for 15 sec at 95°C, annealing for 1 min at 60°C, and extension for 1 min at 72°C). The final extension segment at 72°C was prolonged to 10 min. PCR products were separated using 2.0% agarose gel in Tris-Acetic acid-EDTA buffer (TAE 40.0 mM Tris-base, 20.0 mM Acetic acid, 1.0 mM EDTA, pH 8.0) stained with 10 ng/mL ethidium bromide and left for 45 min in a TAE-filled electrophoresis chamber supplied with 100 V (Agarose gel electrophoresis; AGE). Amplicon size was estimated using the molecular weight ladders. Urine supplemented with adult *S. japonicum* DNA and urine of patients from non-endemic areas were used as positive and negative controls, respectively. A positive sample would show the presence of a single band with the expected molecular weight (254 base pairs) (24).

The closed-tube LAMP assay targeting the 28s rDNA gene was prepared in 25 μL total volume consisting of 1x Isothermal Amplification Buffer (New England Biolabs), 6 mM MgSO_4_ (New England Biolabs), 1 mM each dNTPs (New England Biolabs), 0.8 M Betaine (Sigma-Aldrich), 0.2 μM each of Sj28F3 (5′-GCTTTGTCCTTCGGGCATTA-3′) and Sj28B3 (5′-GGTTTCGTAACGCCCAATGA-3′) primers, 1.6 μM each of Sj28FIP (5′-ACGCAACTGCCAACGTGACATACTGGTCGGCT TGTTACTAGC-3′) and Sj28BIP (5′-TGGTAGACGATCCACCTGACCCCTCGCGCACA TGTTAAACTC-3′) primers, 8 U *Bst* 2.0 WarmStart DNA Polymerase (New England Biolabs), 1x GelGreen (Sigma-Aldrich), and 1 μL gDNA sample. The reaction mixtures were incubated at 64°C for 60 min and followed by an inactivation step at 95°C for 5 min in an ABI thermal cycler. After incubation, the closed reaction tubes were visually assessed with the naked eye by two observers under a 254 nm UV transilluminator. LAMP products showing fluorescence were considered positives (25). To prevent cross contamination between samples, DNA extraction, reagent preparation, PCR and LAMP reactions, and product visualization were performed in separate rooms. Aseptic techniques and protocols to reduce DNA contamination in the laboratory were strictly adhered during processing. A closed-tube LAMP assay was used, which lessened risk of carry-over contamination of amplicons (26).

#### Ultrasound and Clinical Evaluation

A portable Vscan Pocket Ultrasound (GE Healthcare, United Kingdom) equipment was used in the study. US procedure followed the protocol of Ohmae etal. (27) with few modifications based on WHO recommendations (27, 28). All US examinations and interpretation at all time points were performed by two trained personnel to lessen inter-reader variability. Liver images were obtained by substernal, subcostal, intracostal, and sagittal scans, with the patients lying on their backs. Spleen images were taken by intracostal scans with the patients lying on their left sides. The following measurements were taken left liver lobe length in a longitudinal section left parasternal border; right liver lobe length based on the maximum oblique diameter using the right anterior axillary view; portal vein diameter in a right oblique view along the axis of the vessel with the internal diameter of the portal vein at its entry point into the liver; and spleen length in a left oblique view with the maximum length in a section measured through splenic hilus. Height-specific normal values for liver and spleen organometries were based on reference measurements among the healthy Chinese population due to the unavailability of data among healthy Filipinos. This reference measurement was chosen as the source study followed US protocol used in research involving schistosomiasis-induced liver pathology. Hepatosplenic enlargement and atrophy were defined as 2 standard deviations (SD) above and below the mean, respectively. Portal vein wall thickness was expressed as external diameter minus the lumen diameter. Portal vein dilatation and portal vein wall thickening were defined as 2 SD above the mean (29). US images of the liver were classified into three types based on the observed echogenic pattern and the thickness of the portal vein wall: 1.) within normal limits (WNL) or type 0 shows no echogenic patterns and absence of portal vein wall thickening; 2.) mild fibrosis or types 1 and 2 shows linear or tubular echogenic bands with mild, moderate, or severe echogenic thickening (6mm) of the portal vein wall; and 3.) network pattern or type 3 shows septal formation into regular geometric blocks, with more than three blocks surrounded by high echogenic bands (20). Analyses of images were performed in the Philippines.

### Statistical Analyses

Disease positivity in each village was determined based on the results of the various examinations. The intensity of infection was calculated using the arithmetic mean of EPG in the six fecal smears. Results were presented as mean ± standard deviation (SD) for quantitative data and as percentages for qualitative data. Comparisons between groups for quantitative data were performed using the Chi-square test. One-way ANOVA was used for within-group comparisons for continuous variables. Sensitivity, specificity, positive predictive value, and negative predictive value were computed to assess diagnostic performances using a composite reference standard as recommended by WHO/TDR (30). The degree of agreement of the individual tests to the composite standard was assessed using Kappa statistics and classified according to the published guidelines (31). Double-entered data stored in EpiData 3.1 were analyzed using GraphPad Prism version 9 for Mac. A *p*-value of < 0.05 was considered statistically significant.

## Results

**Supplementary Table 1** summarizes the pertinent socio-demographic and clinical characteristics of participants in the cross-sectional study. Among the dependent variables, being female (*p* < 0.0438), having no formal education (Chi-square test, χ^2^ = 21.50, df = 3, *p* < 0.0001), having a farming-related occupation (*p* < 0.0001), and inconsistent participation (missing at least one dose of praziquantel) in MDA in the last 2 years (*p* < 0.0002) were significantly associated with stool positivity. Other variables such as living or working near snail colonies (*p* = 0.8799), ownership of latrine (*p* = 0.8535), presence of symptoms suggestive of schistosomiasis (*p* = 0.2810), history of schistosomiasis based on stool examination (*p* = 0.1351), previous abdominal US (*p* = 0.6705), and reactivity to HBsAg (*p* = 0.7172) were not significantly correlated with stool positivity.

**Table 1** presents the estimated positivity rate of schistosomiasis at 95% CI based on 3 K-K, SEA ELISA, and the hepatosplenic US. SEA ELISA reported the highest proportion of cases (41.6%) followed by 3 K-K (27.4%), with the US detecting the least cases (23.7%). The number of cases detected by SEA ELISA was higher than the other tests in all endemic barangays. Among the study sites, the highest proportion of stool positives was recorded in Dita at 35.5% (95% CI [29.2, 43.0]), while San Roque had the lowest proportion of stool positive individuals at 18.5% (95% CI [11.7, 29.2]).

**Table 1:** Positivity of schistosomiasis at baseline based on conventional diagnostic tests.

Study Sites		Schistosomiasis Positivity (%)					
Municipality	Barangay	3 K-K		SEA ELISA		US	
		N	%(95% CI)	N	%(95% CI)	N	%(95% CI)
Alangalang	Bugho n = 159	45	28.3 (22.0-36.2)	85	53.5 (46.2 – 61.8)	46	28.9 (22.7 – 36.9)
	SAF n = 139	37	26.6 (20.2-35.1)	69	49.6 (42.0 – 58.7)	23	16.5 (11.4 – 24.0)
Palo	Cangumbang n = 138	28	20.3 (14.6-28.2)	42	30.4 (23.6 – 39.2)	38	27.5 (21.0 – 36.1)
	Tacurangan n = 73	24	32.9 (23.7-45.6)	42	57.5 (47.2 – 70.1)	9	12.3 (6.7 – 22.7)
Julita	Dita n = 189	67	35.5 (29.2-43.0)	79	41.8 (35.3 – 49.5)	52	27.5 (21.8 – 34.7)
	Calbasag n = 114	29	25.4 (18.6-34.8)	31	27.2 (20.1 – 36.7)	31	27.2 (20.1 – 36.7)
Sta Fe	San Juan n = 87	24	27.6 (19.6-38.8)	37	42.5 (33.3 – 54.3)	23	26.4 (18.6 – 37.5)
	San Roque n = 81	15	18.5 (11.7-29.2)	23	28.4 (20.0 – 40.1)	10	12.3 (6.9 – 22.1)
TOTAL (n = 980)		269	27.4 (24.8-30.4)	408	41.6 (38.7 – 44.8)	232	23.7 (21.2 – 26.5)

3 K-K, Three stool Kato-Katz; (SEA) ELISA, Soluble Egg Antigen; US, Ultrasound.

Among the 980 participants, parenchymal fibrosis of the liver was the most common US pathology, observed in 232 patients (23.7%). Other US findings consistent with schistosomiasis were splenomegaly in 81 patients (8.3%), hepatomegaly in 69 patients (7.0%), and portal vein wall fibrosis in 55 patients (5.6%). Using the Ohmae etal. (27) classification, there were 748 (76.3%), 101 (10.3%), and 131 (13.4%) with type 0 (no fibrosis), types 1-2 (mild fibrosis), and type 3 (severe fibrosis) US findings, respectively (27). Most had no clinical manifestations of hepatic disease. Six had signs and symptoms suggestive of decompensated liver disease.

All examinations except for the US were more sensitive than the widely utilized K-K technique, even with repeated fecal sampling. **Table 2** summarizes the diagnostic performance parameters and agreement values with the composite reference standard (CRS) of the various examinations performed on samples from 230 patients (**Supplementary Table 2**). Multiple stool examinations enhanced the sensitivity of K-K from 26.2% (95% CI [16.4, 38.8]) with single stool to 53.8% (95% CI [41.1, 66.1]) and 69.2% (95% CI [56.4, 80.0]) with two and three stools from consecutive days, respectively. Among the SjcDNA nucleic acid amplification test (NAAT)-based detection assays, loop-mediated isothermal amplification (LAMP) PCR using sera had the highest sensitivity at 92.3% (95% CI [82.2, 97.1]) with LAMP consistently identifying more positive cases in both serum and urine samples. Regarding sample type, SjcDNA was detected more in sera than in urine samples. Although POC-CCA had the same sensitivity with three stool K-K, the former had low specificity (69.7%). US (K 0.15, 95% CI [0, 0.31]) had slight agreement while POC-CCA (K 0.34, 95% CI [0.21, 0.47]) and 1 K-K (K 0.33, 95% CI [0.17, 0.50]) had fair agreement. SEA ELISA (K 0.6, 95% CI [0.49, 0.71]), 2 K-K (K 0.62, 95% CI [0.50, 0.75]), and 3 K-K (K 0.76, 95% CI [0.66, 0.86]) had substantial agreement. All the SjcDNA detection assays had an excellent agreement.

**Table 2:** Diagnostic performances of the different schistosomiasis examinations of samples collected from 230 participants using a composite reference standard (CRS).

Examination	Diagnostic Performance				
	Sensitivity (95% CI)	Specificity (95% CI)	PPV (95% CI)	NPV (95% CI)	K
sLAMP	92.3 (82.2-97.1)	100.0 (97.2-100.0)	100.0 (92.5-100.0)	97.1 (92.9-98.9)	0.95
uLAMP	83.1 (71.3-90.9)	100.0 (97.2-100.0)	100.0 (91.7-100.0)	93.8 (88.8-96.7)	0.88
POC-CCA	69.2 (56.4-79.8)	69.7 (62.0-76.5)	47.4 (37.1-57.8)	85.2 (77.8-90.5)	0.34
sPCR	87.7 (76.6-94.2)	100.0 (97.2-100.0)	100.0 (92.1-100.0)	95.4 (90.8-97.8)	0.91
uPCR	80.0 (67.9-88.5)	100.0 (97.2-100.0)	100.0 (91.4-100.0)	92.7 (87.6-95.9)	0.85
3 K-K	69.2 (56.4-80.0)	100.0 (97.2-100.0)	100.0 (90.2-100.0)	89.2 (83.6-93.1)	0.76
2 K-K	53.8 (41.1-66.1)	100.0 (97.2-100.0)	100.0 (87.7-100.0)	84.6 (78.6-89.2)	0.63
1 K-K	26.2 (16.4-38.8)	100.0 (97.2-100.0)	100.0 (77.1-100.0)	77.5 (71.1-82.8)	0.34
SEA ELISA	84.6 (73.1-92.0)	81.8 (74.9-87.2)	64.7 (53.5-74.6)	93.1 (87.3-96.5)	0.61
US	30.8 (20.2-43.6)	83.6 (76.9-88.8)	42.6 (28.6-57.7)	75.4 (68.4-81.3)	0.16

Sn, Sensitivity; Sp, Specificity; PPV, Positive Predictive Value; NPV, Negative Predictive Value; K, Kappa Coefficient; sLAMP, Serum LAMP; uLAMP, Urine LAMP; POC-CCA, Point-of-care circulating cathodic antigen urine test; sPCR, Serum PCR; uPCR, Urine PCR; 3 K-K, Three stool Kato-Katz; 2 K-K, Two stool Kato-Katz; 1 K-K, Single stool Kato-Katz; (SEA) ELISA, Soluble Egg Antigen; US, Ultrasound.

**Figure 3:**
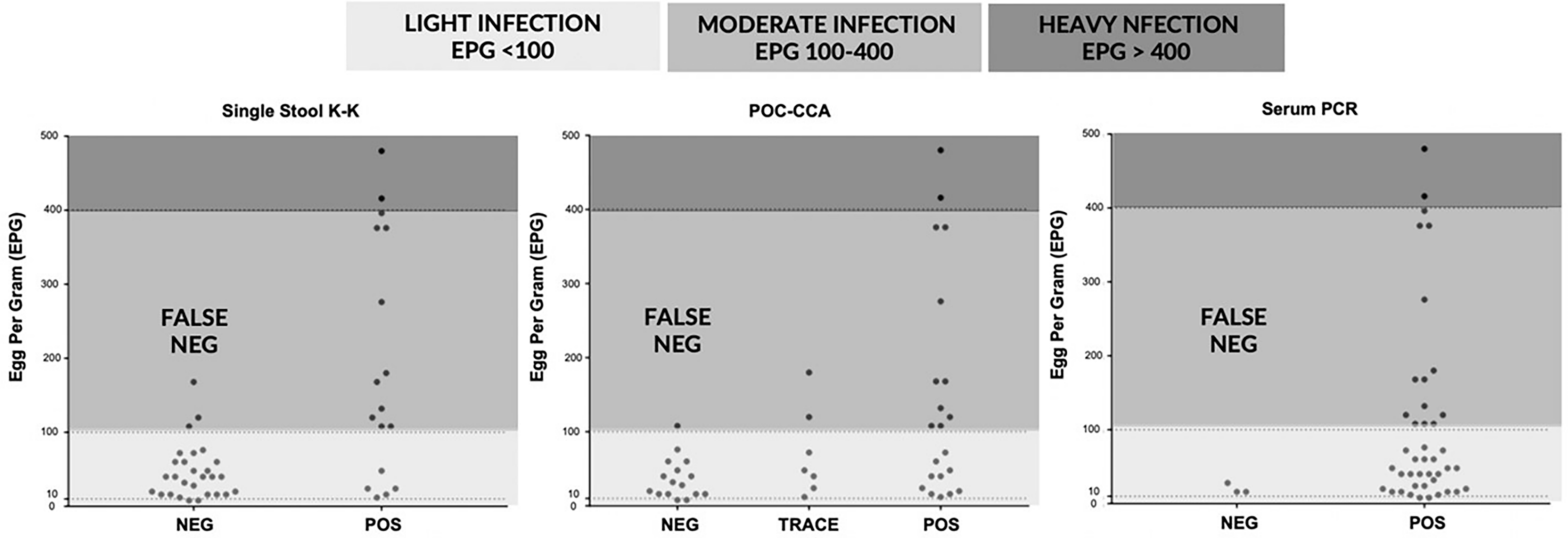
Correlations of egg per gram (EPG) determined by three stool Kato-Katz with results of single stool Kato-Katz, point of care circulating cathodic antigen (POC-CCA) urine test, and serum PCR (n = 45).

**Figure 3** depicts the correlation of EPG based on three stool K-K, an indirect measure of infection intensity, with results of single stool K-K (Spearman’s rho = 0.48, *p* < 0.001), POC-CCA (Spearman’s rho = 0.44, *p* < 0.001), and serum PCR (Spearman’s rho = 0.268, *p* = 0.075). POC-CCA and single stool K-K were more likely to miss diagnosing light infections than the SjcDNA detection assays.

**Figure 4:**
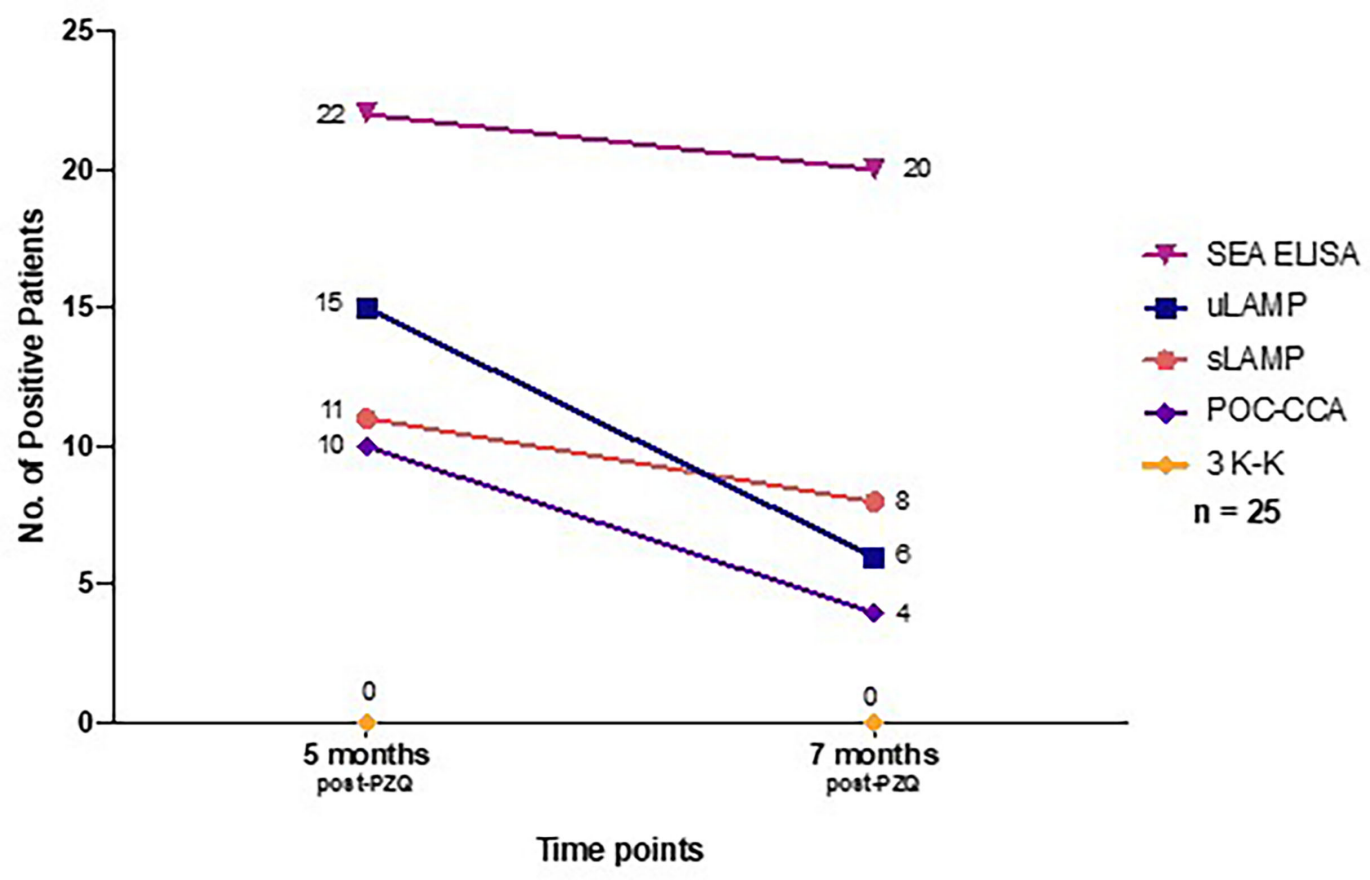
Number of positive patients at 5 months and 7 months post-PZQ treatment based on different diagnostic tests (n = 25). PZQ, Praziquantel; SEA IgG ELISA, Soluble Egg Antigen; uLAMP, Urine LAMP; sLAMP, Serum LAMP; POC-CCA, Point-of-care circulating cathodic antigen urine test; 3 K-K, Three consecutive days stool Kato-Katz.

Among the 45 stool positive cases in the diagnostic study, 25 patients positive in SEA ELISA, urine LAMP (uLAMP), serum LAMP (sLAMP), POC-CCA, and 3 K-K at baseline were selected to participate in the post-chemotherapy study done at 5 and 7 months after PZQ treatment (**Table 3**). All 25 cases had negative 3 K-K at 5 and 7 months post-PZQ. The number of positive patients in sLAMP, uLAMP, and POC-CCA decreased at 5 and 7 months from 11 to 8, 15 to 6, and 10 to 4, respectively (**Figure 4**). SEA ELISA had a low rate of conversion to negativity of the results, with only five patients (16.1%) being non-reactive even at seven months post-PZQ.

**Table 3:** Negative conversion of the different diagnostic examinations in 25 patients 5 and 7 months after completion of praziquantel treatment.

Periods after treatment	N of examined cases	Negative conversion, N (%)				
		3 K-K	sLAMP	uLAMP	POC-CCA	SEA ELISA
5 months	25	25 (100)	14 (56.0)	10 (40.0)	15 (60.0)	3 (9.7)
7 months	25	25 (100)	17 (68.0)	19 (76.0)	21 (84.0)	5 (16.1)

3 K-K, Three stool Kato-Katz; sLAMP, Serum LAMP; uLAMP, Urine LAMP; POC-CCA, Point-of-care circulating cathodic antigen urine test; (SEA) ELISA, Soluble Egg Antigen.

## Discussion

Despite the multiple studies showing its poor sensitivity in areas of low prevalence and among patients with light infection, K-K stool examination remains the primary technique used in endemic regions for different diagnostic purposes (32–34). The low sensitivity of K-K examination using single stool has erroneously resulted in reports of the apparent absence of cases in some endemic villages in the Philippines, with some regional schistosomiasis coordinators requesting to stop mass drug administration (MDA) in their localities (8). Single stool K-K detected *S. japonicum* eggs in most patients with moderate to heavy infection but missed the diagnosis of most individuals with light infection (**Figure 3**). Although repeated fecal collection and examination improved diagnostic sensitivity, this procedure might compromise the inherent simplicity and low cost of K-K (35).

SjcDNA was detected in more sera than urine samples despite using an additional DNA concentration technique for urine, possibly due to unequal distribution of SjcDNA in the two body fluids. The mature adult schistosomes lodged in the portal or mesenteric veins and the migrating schistosomula are in direct contact with the venous blood, resulting in higher levels of SjcDNA in serum than in urine (36). Compared with tests using serum, urine-based diagnostics are generally easier, less invasive, and result in better community compliance. Using bigger volumes of urine might increase the yield of urine SjcDNA detection (9).

LAMP assay detected more individuals with SjcDNA in their sera and urine samples than conventional PCR. This is consistent with previous reports showing LAMP to be more sensitive than endpoint PCR in detecting schistosome circulating DNA in body fluids (37). Aside from better diagnostic performance, LAMP is more field applicable than PCR as the former does not require a thermal cycler and electrophoresis set-up. Although the currently applied LAMP system is not yet field-deployable due to the need for DNA extraction, modifications can be done to enable on-site LAMP analyses, such as using simpler DNA extraction protocols with shorter incubation time and lesser need for expensive equipment.

Similar to previous studies, the different diagnostic tests were compared to a composite reference standard, which is used in the absence of a single suitable “gold standard” (38–41). Despite using duplicate smears from multiple stools collected from different days, Kato-Katz is still an imperfect test to diagnose intestinal schistosomiasis. POC-CCA showed a sensitivity of 69.2%, which is superior to single and two stool K-K, comparable to three stool K-K, and inferior to the SjcDNA detection assays. Similar diagnostic sensitivity of POC-CCA against multiple stool K-K was reported in studies done in Asia (15), Africa (42), and South America (43, 44). An important limitation of POC-CCA shown in this study is its high number of false positives, resulting in low specificity (69.7%) and only fair agreement (κ = 0.34) with the CRS (**Table 2**). Previous studies have recognized that the false positives of POC-CCA might be due to its cross-reactivity with pregnancy, conditions associated with proteinuria and hematuria, and other helminthic diseases such as opisthorchiasis and hookworm infection (45, 46). Additional information is necessary to elucidate the diagnostic implications of trace results and co-infection when applying POC-CCA for Intestinal schistosomiasis.

The other diagnostic test available, albeit not widely used in selected endemic areas of the Philippines are antibody testing using the COPT and US. US is a reliable and field applicable tool for evaluating the severity of schistosome egg-induced hepatosplenic abnormalities. US is more useful in establishing the presence of chronic disease with the presence of liver and spleen morbidities (33, 47). In this study, 20.4% were positive based on US findings. The presence of liver abnormalities based on US was higher than stool positivity, which was similarly reported in other areas of the Philippines (20). US abnormality usually manifests as hepatic fibrosis without overt clinical signs of liver disease. Even in the absence of active schistosomiasis infection, patients with this residual chronic liver pathology are at risk of developing more serious forms of liver disease from other communicable and non-communicable causes (48).

This study also assessed the ability of the different examinations to record treatment effectiveness. After completion of the PZQ regimen, all 25 cases enrolled in the treatment effectiveness study had negative stool examination at 5 and 7 months. The negative seroconversion rate of SEA ELISA remained low, while for the serum and urine LAMP assays and POC-CCA, their conversion rates to negativity of the results increased at 5 and 7 months. The remaining LAMP and POC-CCA positive cases might represent one of the following scenarios: (1) delayed clearance of the SjcDNA and CCA after successful treatment, (2) incomplete elimination of schistosomes, or (3) re-infection. Post-treatment studies using animal models have shown that SjcDNA was undetectable 6-8 weeks after PZQ exposure (37, 49, 50). Since patients in this study are constantly exposed to contaminated water bodies and might be chronically infected, delayed clearance of the SjcDNA might be due to the slower release of DNA from dead eggs deposited in the patient’s tissues (12).

A significant limitation of our study is that the CRS used to estimate the diagnostic performance of the different tests consists of in-house or non-commercial assays. Although the conventional PCR and LAMP assays were based on previous publications and the current study strictly adhered to their written protocols, there are still critical procedural variations that can affect the performance of these tests. Since there are no widely available commercial tests for intestinal schistosomiasis in the Philippines, the study still opted to include the in-house molecular tests in the CRS. The purposive sampling of high-risk individuals enrolled in the cohort is another limitation. The positivity using the different tests cannot be the basis for estimating schistosomiasis prevalence and should be cautiously interpreted.

## Conclusion

In conclusion, this study provides further evidence that single stool K-K remains the only diagnostic test available in most endemic areas in the Philippines. It had low sensitivity and failed to identify most patients with light infection. Newer diagnostic tests such as SjcDNA detection assay and POC-CCA urine test were more sensitive than stool microscopy in detecting schistosomiasis. On the other hand, US was less sensitive than the widely utilized K-K technique in diagnosing schistosomiasis. However, it is more helpful in establishing the presence of chronic disease with the presence of liver and spleen morbidities. This study emphasizes the need to revisit the use of single stool K-K in the surveillance and case detection of schistosomiasis in endemic areas of the Philippines. The availability of advanced and more sensitive diagnostic tests will help better control, prevent, and eliminate schistosomiasis in the country.

## Data Availability Statement

The original contributions presented in the study are included in the article/**Supplementary Material**. Further inquiries can be directed to the corresponding authors.

## Ethics Statement

The studies involving human participants were reviewed and approved by University of the Philippines Manila Research Ethics Board UPMREB Code 2017-369-01. The patients/participants provided their written informed consent to participate in this study.

## Author Contributions

IT, LL, PM, and MiK formulated the research objectives and prepared the study protocol. IT, RF, RR, YC, and MK performed participant examination, data acquisition, and sample collection in the field. IT, RF, MOS, MS, MaK, YC, and MiK performed the different diagnostic tests in the field and in the laboratory. IT and OT performed data analysis and prepared the draft manuscript. LL and IF reviewed and edited the manuscript. All authors contributed to the article and approved the submitted version.

## Funding

This study was supported by the Department of Science and Technology Philippine Council for Health Research and Development (DOST PCHRD) and Japan Society for the Promotion of Science (JSPS) Grant-in-Aid for Research (KAKENHI) Project Number: 15K08445 awarded to MK.

## Conflict of Interest

The authors declare that the research was conducted in the absence of any commercial or financial relationships that could be construed as a potential conflict of interest.

## Publisher’s Note

All claims expressed in this article are solely those of the authors and do not necessarily represent those of their affiliated organizations, or those of the publisher, the editors and the reviewers. Any product that may be evaluated in this article, or claim that may be made by its manufacturer, is not guaranteed or endorsed by the publisher.
