# Supplementary material for: Diagnostic Performance of Parasitological, Immunological, Molecular, and Ultrasonographic Tests in Diagnosing Intestinal Schistosomiasis in Fieldworkers From Endemic Municipalities in the Philippines
Source: Front Immunol. 2022 Jun 14;13:899311. doi: 10.3389/fimmu.2022.899311 (PMC9237846; doi:10.3389/fimmu.2022.899311)
Supplement: Supplementary file 1 [file DataSheet_1.docx]

**Supplementary Table**

#### **Table 1****.** Pertinent characteristics of participants in the initial cross-sectional survey.

| **Variable** | **Participants (N=980)**  **N (%)** | **Stool positives (N=269)**  **N (%)^a^** | ***p*-value^b^** |
| --- | --- | --- | --- |
| **Sex** | | | |
| Female | 580 (59.2) | 151 (22.5) | < 0.04^S^ |
| Male | 423 (40.8) | 118 (27.9) |  |
| **Age in years** | | | |
| Range | 18-73 | - | - |
| Mean (SD) | 34.0 (9.9) | - |  |
| Median | 38 | - |  |
| **Highest educational attainment^a^** | | | |
| None | 101 (10.3) | 47 (35.9) | < 0.01^S^ |
| Elementary school | 590 (60.2) | 158 (24.8) |  |
| High school | 234 (23.9) | 46 (17.5) |  |
| College or more | 55 (5.6) | 8 (12.31) |  |
| **Occupation** | | | |
| Farming-related | 727 (74.2) | 245 (30.5) | < 0.01 ^S^ |
| Not farming-related | 253 (25.8) | 24 (8.2) |  |
| **Living or working near snail colonies** | | | |
| Yes | 692 (70.6) | 186 (24.7) | 0.88 ^NS^ |
| No | 288 (29.4) | 83 (24.2) |  |
| **Ownership of latrine at house** | | | |
| Yes | 942 (96.1) | 260 (24.7) | 0.85 ^NS^ |
| No | 38 (3.9) | 9 (22.0) |  |
| **Currently with symptoms related to schistosomiasis** | | | |
| Yes | 183 (18.7) | 44 (21.6) | 0.28 ^NS^ |
| No | 797 (81.3) | 225 (25.3) |  |
| **History of schistosomiasis based on stool examination** | | | |
| Yes | 323 (33.0) | 78 (21.7) | 0.14^NS^ |
| No | 657 (67.0) | 191 (25.6) |  |
| **Regular participation in MDA for the past 2 years** | | | |
| Yes | 534 (54.5) | 101 (16.2) | < 0.01 ^S^ |
| No | 446 (45.5) | 145 (30.7) |  |
| **Previous abdominal US** | | | |
| Yes | 24 (2.5) | 6 (20.0) | 0.67 ^NS^ |
| No | 951 (97.0) | 263 (24.7) |  |
| **HBsAg serology** | | | |
| Reactive (positive) | 82 (9.4) | 23 (22.6) | 0.72 ^NS^ |
| Non-reactive (negative) | 898 (91.6) | 246 (24.8) |  |
| **Anti-HCV serology** | | | |
| Reactive (positive) | 4 (0.4) | 0 (0) | - |
| Non-reactive (negative) | 976 (99.6) | 269 (24.7) |  |

**Note:** ^a^ positive in at least 1 out of 6 stool Kato-Katz smears, ^b^ based on Chi-square or Fisher exact test for contingency table analyses, ^S^ statistically significant at p-value < 0.05, ^NS^ not statistically significant at p-value ≥ 0.05; **Abbreviations:** Mass Drug Administration (MDA), Hepatitis B Surface Antigen (HBsAg), Anti-hepatitis C virus IgG (Anti-HCV), Ultrasound (US)

**Supplementary Table 2**. Positivity of schistosomiasis based on different diagnostic examinations among the participants.

| **Study site** |  |  | **Schistosomiasis Cases** | | | | | | | | | |
| --- | --- | --- | --- | --- | --- | --- | --- | --- | --- | --- | --- | --- |
|  | **CRS** | **sLAMP** | | **uLAMP** | **CCA** | **sPCR** | **uPCR** | **3 K-K** | **2 K-K** | **1 K-K** | **SEA** | **UTZ** |
|  | **No. (%)** | **No. (%)** | | **No. (%)** | **No. (%)** | **No. (%)** | **No. (%)** | **No. (%)** | **No. (%)** | **No. (%)** | **No. (%)** | **No. (%)** |
| Bugho (LE)  n = 77 | 15 (19.5) | 12 (15.6) | | 10 (13.0) | 17 (22.1) | 12 (15.6) | 9 (11.7) | 11 (14.3) | 8 (10.4) | 2 (2.6) | 25 (32.5) | 16 (20.8) |
| Cangumbang (ME) n = 74 | 21 (28.4) | 18 (24.3) | | 17 (23.0) | 33 (44.6) | 15 (20.3) | 17 (23.0) | 14 (18.9) | 10 (13.5) | 3 (4.1) | 28 (37.8) | 14 (18.9) |
| Dita (HE)  n =79 | 34 (43.0) | 30 (38.0) | | 27 (34.2) | 45 (57.0) | 30 (38.0) | 26 (32.9) | 20 (25.3) | 17 (21.5) | 12 (15.5) | 32 (40.5) | 17 (21.5) |
| **TOTAL** n = 230 | 65 (28.3) | 60 (26.1) | | 54 (23.5) | 95 (41.3) | 57 (24.8) | 52 (22.6) | 45 (19.6) | 35 (15.2) | 17 (7.4) | 85 (37.0) | 47 (20.4) |

**Abbreviations**: LE: low endemicity; ME: moderate endemicity: HE: high endemicity; CR: composite reference standard; sPCR: serum PCR; uPCR: urine PCR; sLAMP: serum LAMP; uLAMP: urine LAMP; 3 K-K: three stool Kato-Katz; 2 K-K: two stool Kato-Katz; 1 K-K single stool Kato-Katz; CCA: point-of-care circulating cathodic antigen; SEA: soluble egg antigen ELISA; UTZ: ultrasound changes
